# Supplementary material for: Osmotic Stress Responses, Cell Wall Integrity, and Conidiation Are Regulated by a Histidine Kinase Sensor in Trichoderma atroviride
Source: J Fungi (Basel). 2023 Sep 16;9(9):939. doi: 10.3390/jof9090939 (PMC10532544; doi:10.3390/jof9090939)
Supplement: Supplementary file 1 [file jof-09-00939-s001.zip › jof-2569194-supplementary.pdf]

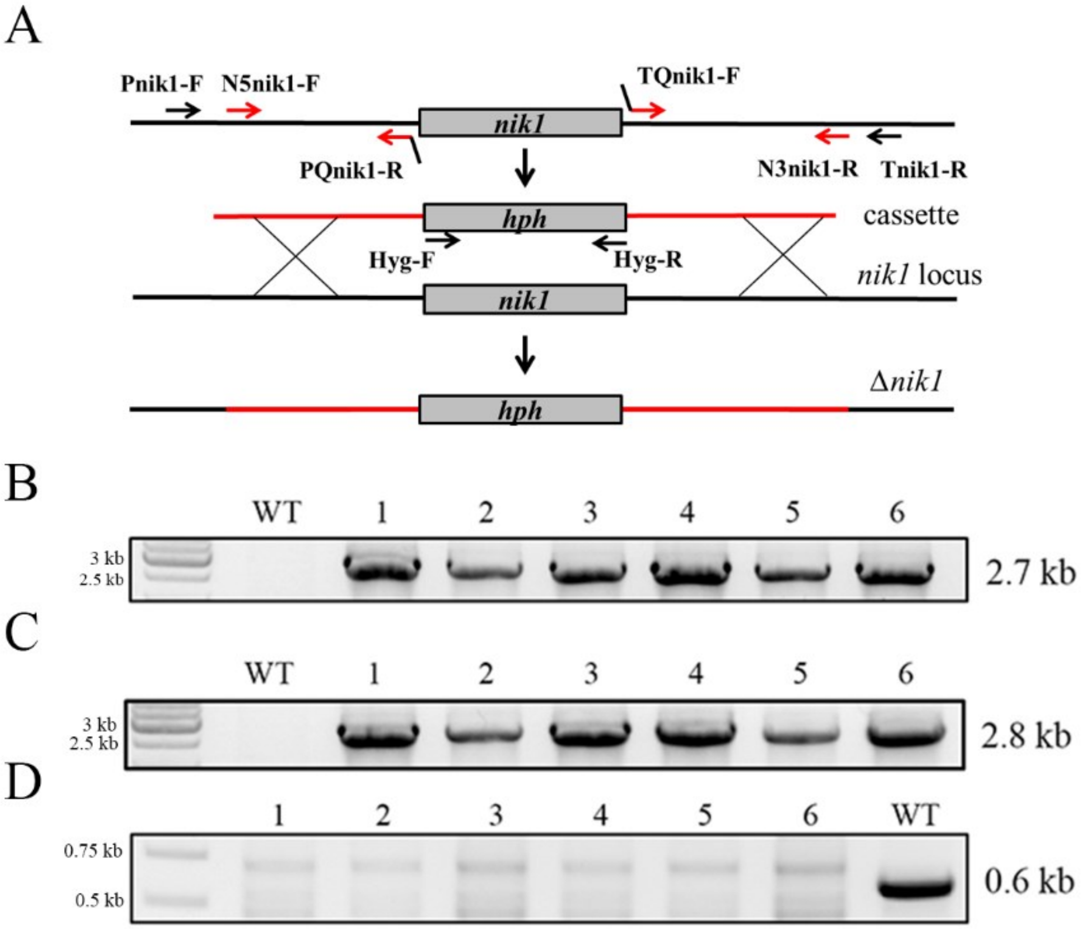

Figure S1. Deletion of *nik1* gene. **A.** Schematic diagram of the *nik1* gene replacement by the *hph* selectable marker. **B-D**. PCR to confirm the *nik1* gene replacement in Δ*nik1* strains. Pnik1-F ─ Hyg-R primers were used to amplify the 5’ region - *hph* gene (**B**). Hyg-F – Tnik1-R primers amplified the *hph* - 3’ region (**C**). 1nik1-F – 3nik1-F primers amplified a fragment of the *nik1* ORF (**D**).

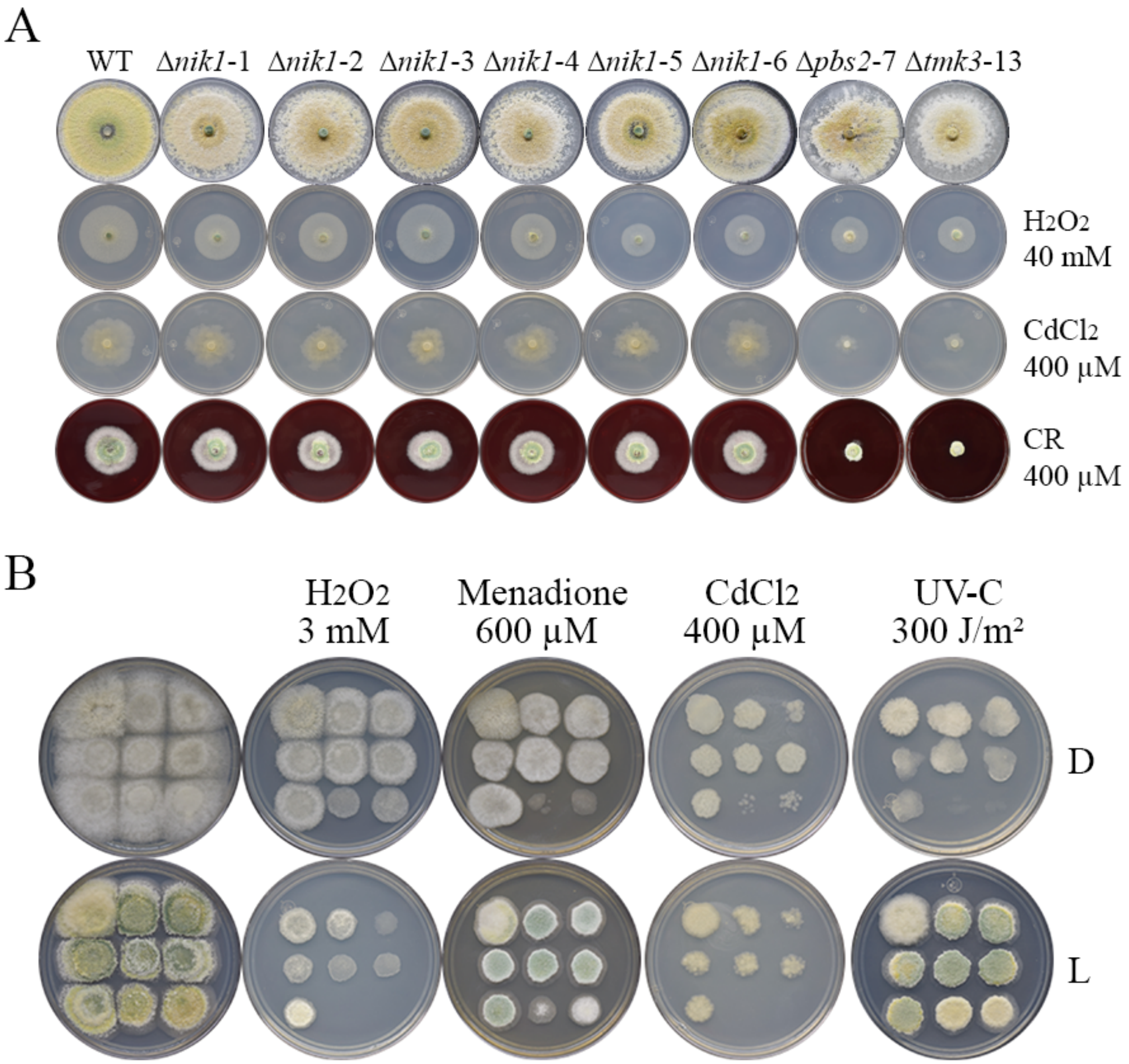

Figure S2. Response to oxidative stress, cadmium, and UV-light in Δ*nik1* strains. **A.** Tolerance to oxidative stress, cadmium and Congo red in mycelia of WT, Δ*nik1*, Δ*pbs*2 and Δ*tmk*3 strains. H_2O_2, $CdCl_2$ and Congo red were added to PDA media at the indicated concentrations. Strains were incubated at 27°C for four days in constant white-light and pictures were taken. **B.** Tolerance to oxidative stress, cadmium and UV-light in conidia of WT, Δ*nik1*, Δ*pbs*2 and Δ*tmk*3 strains. Drops of 500 conidia were inoculated of the following strains (top-left to bottom-right): WT, Δ*nik1*-1, -2, -3, -4, -5, -6, Δ*pbs*2-7, and Δ*tmk*3-13. Strains were incubated at 27°C for four days in darkness (D) or constant white-light (L) and pictures were taken. A Stratalinker 2400 UV Crosslinker was used to emit UV irradiation. Assays were performed in triplicate.

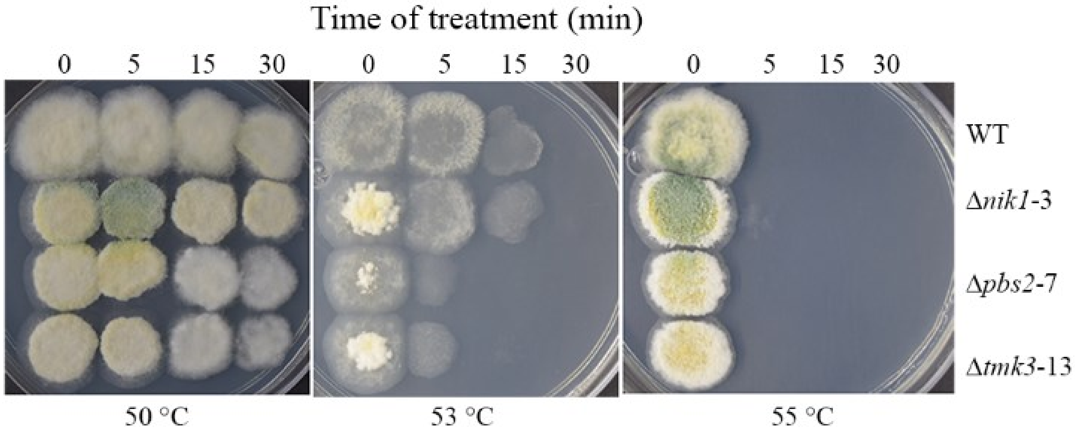

Figure S3. Thermal-shock resistance in Δ*nik1* strains. A conidial suspension (100 conidia μl^{-1}) of WT, Δ*nik1*-3, *pbs*2-7, and Δ*tmk*3-13 strains was incubated in a Thermoblock at the indicated temperature during the indicated times. Then, drops of 500 conidia were inoculated on PDA plates plus Triton X-100 0.5 % and incubated at 27 °C by 4 days, and pictures were taken. The assay was performed in triplicate.

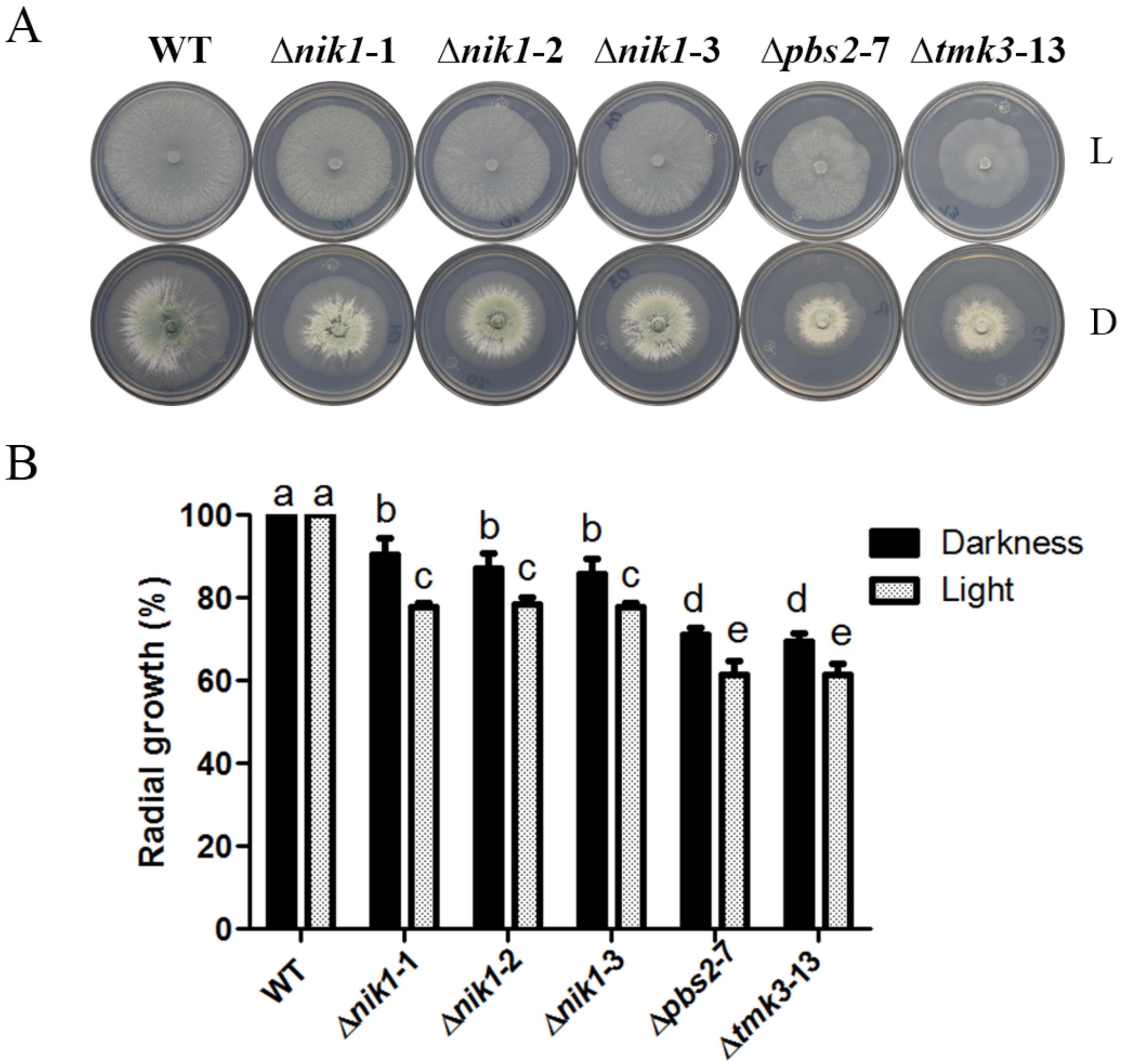

Figure S4. Radial growth of Δ*nik1* strains. **A.** WT, Δ*nik1*, Δ*pbs*2 and Δ*tmk*3 strains were inoculated on PDA plates at 27°C for 48 h in constant white-light (L) or darkness (D). **B.** After 48 h, pictures were taken and radial growth was measured in triplicate using the Image J software (version 1.52a). The graph shows the averages ± the standard deviation of three independent experiments, and analyzed with the Tukey-Kramer method (α=0.05). Different letters on the bars represent significant differences.

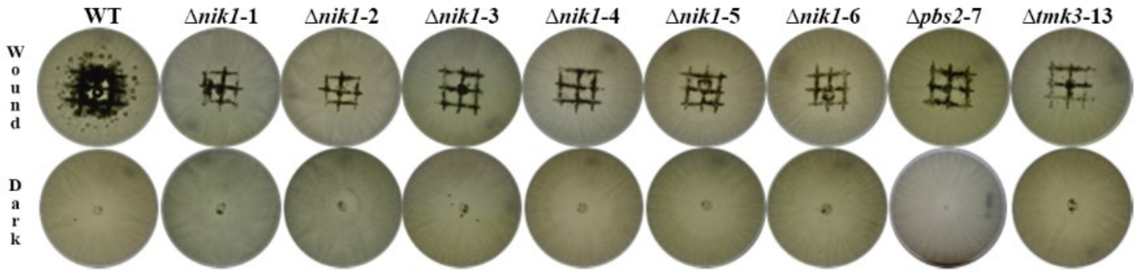

Figure S5. Wound response assay in Δ*nik1* strains. Mycelial plugs of the indicated strains were inoculated on PDA plates and incubated at 27°C in darkness. After 48 h, mycelia of the WT and mutant strains were damaged with a sterile scalpel performing six cuts (Wound), incubated for an additional 48 h in the dark, and pictures were taken. Strains maintained in complete darkness (Dark) were used as control.
